# Lipid coated liquid crystal droplets for the on-chip detection of antimicrobial peptides†
†The full data sets are available at doi.org/10.5518/466.
‡
‡Electronic supplementary information (ESI) available. See DOI: 10.1039/c8lc01291a


**DOI:** 10.1039/c8lc01291a

**Published:** 2019-02-20

**Authors:** Peng Bao, Daniel A. Paterson, Patrick L. Harrison, Keith Miller, Sally Peyman, J. Cliff Jones, Jonathan Sandoe, Stephen D. Evans, Richard J. Bushby, Helen F. Gleeson

**Affiliations:** a School of Physics and Astronomy , University of Leeds , Leeds , UK . Email: s.d.evans@leeds.ac.uk ; Email: r.j.bushby@leeds.ac.uk ; Email: h.f.gleeson@leeds.ac.uk; b Department of Chemistry and Biochemistry , University of Hull , UK; c Biomolecular Research Centre , Sheffield Hallam University , Sheffield , UK; d Leeds Institute of Biomedical & Clinical Science , University of Leeds , Leeds , UK

## Abstract

We describe a novel biosensor based on phospholipid-coated nematic liquid crystal (LC) droplets and demonstrate the detection of Smp43, a model antimicrobial peptide (AMP) from the venom of North African scorpion *Scorpio maurus palmatus*. Mono-disperse lipid-coated LC droplets of diameter 16.7 ± 0.2 μm were generated using PDMS microfluidic devices with a flow-focusing configuration and were the target for AMPs. The droplets were trapped in a bespoke microfluidic trap structure and were simultaneously treated with Smp43 at gradient concentrations in six different chambers. The disruption of the lipid monolayer by the Smp43 was detected (<6 μM) at concentrations well within its biologically active range, indicated by a dramatic change in the appearance of the droplets associated with the transition from a typical radial configuration to a bipolar configuration, which is readily observed by polarizing microscopy. This suggests the system has feasibility as a drug-discovery screening tool. Further, compared to previously reported LC droplet biosensors, this LC droplet biosensor with a lipid coating is more biologically relevant and its ease of use in detecting membrane-related biological processes and interactions has the potential for development as a reliable, low-cost and disposable point of care diagnostic tool.

## Abstract 2

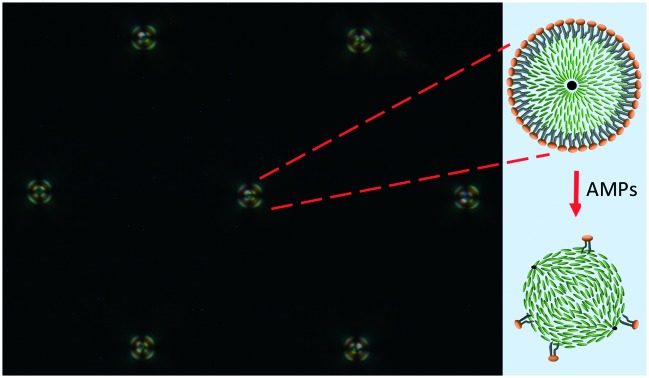
Lipid coated liquid crystal droplets have been trapped in a novel trap structure for the on-chip detection of a model antimicrobial peptide – Smp43, an α-helical peptide from Scorpion Venom.

## 1 Introduction

For many decades, liquid crystal (LC) materials have been widely used in industry for LC display (LCD) applications, due to their well-known electrooptic modulation effects. More recently, LC materials have shown great potential in the field of biosensors.^1^–^6^ Two main forms of LC crystal biosensors have been demonstrated: those based on the interaction of LCs with chemically-modified solid interfaces; and LC biosensors that incorporate a LC/aqueous interface (this class includes both LC thin films and droplets).^1^–^6^ They have been demonstrated for the successful detection of surfactants, lipids, heavy metals, glucose, enzymes, volatile organic compounds, DNAs and proteins, bacteria/mammalian cells and antigens.^1^–^6^ Recently, LC biosensors have also been demonstrated useful for the detection of cationic antimicrobial peptides (AMPs).^7^

AMPs are of interest due to their potential as novel antibiotics, based on their selectivity for prokaryotes and their membrane-disruptive mechanisms for which microbes have little natural resistance.^8^–^10^ As a class of molecules, AMPs have proven to have activity against a remarkably wide range of clinically relevant pathogens including both Gram classes of bacteria, enveloped viruses, and fungi, along with substantial anticancer properties.^11^ This selectivity is typically due to the electrostatic attraction between a cationic AMP and the negatively charged prokaryotic phospholipid membrane, which contains a higher proportion of phosphatidylglycerol and cardiolipin compared with mammalian membranes; these contain a higher proportion of zwitterionic phospholipids such as phosphatidylcholine & phosphatidylethanolamine.^12^ To evaluate the membrane active mechanism of AMPs, the interaction between AMPs and phospholipids has been studied by a variety of biophysical techniques including surface plasmon resonance (SPR), nuclear magnetic resonance (NMR) spectroscopy, fluorescent microscopy, Raman and CD optical spectroscopy and atomic force microscopy (AFM).^13^,^14^ Recently, we have studied the interaction of Smp43, an α-helical peptide with a helical-hinge-helical topology isolated from North Africa scorpion venom, with planar lipid bilayers at the nanoscale using fast scan AFM.^15^ Smp43 was shown to be selectively adsorbed onto atypical prokaryotic mimicking membranes with lipid removal proceeding in a highly branched fashion termed ‘diffusion limited disruption’. The same process observed on mammalian mimicking membranes disruption was severely retarded. There are a number of bacterial toxins that disrupt lipid membranes and thus the membrane-disrupting AMP was viewed as a model agent to establish the principle for the detection of these agents and hence for the presence of the bacteria themselves.

There is an interesting diversity in the approaches using LC materials to detect biomolecular interactions at a LC/aqueous interface in thin film geometries, especially with lipid decoration.^2^,^16^–^18^ As natural amplifiers, LCs can transduce and amplify the changes at the interface induced by a range of interactions and show visible optical signals under a polarized microscope.^19^,^20^ With lipid-decoration, the LC molecules near the interface adopt a homeotropic alignment, attributed to the molecular interdigitation of the acyl tails of the lipids and the mesogens.^2^ Lipid-decorated LC/aqueous interfaces have been used to report protein binding and enzymatic events of phospholipase A2 and beta-bungarotoxin.^21^,^22^ This approach was also used to study the spatial organization and dynamics of protein networks, as well as the interaction of cationic AMPs with anionic lipids.^7^,^13^ To date there have been no reports of LC biosensors based on lipid-coated liquid crystal droplets^8^ despite the some potential advantages, which include ease of manufacture and low cost.

Microfluidic devices have been used extensively in the field of synthetic chemistry and life science in the past twenty years.^23^–^26^ This technology has shown great potential in single droplet or single cell studies.^27^ Many kinds of traps have been demonstrated for the *in situ* study of single cells or droplets in microfluidic devices.^25^,^28^–^30^ These not only enable the localization of the droplets, but also offer the possibility of quick buffer exchanges and *in situ* analysis.^30^ In recent years, the application of microfluidic devices in liquid crystal biosensor research has emerged.^31^,^32^ For example, a highly reproducible method to form uniform LC thin films in a microfluidic channel has been demonstrated and used for the successful detection of analytes in the aqueous phase.^31^ However, the combination of generation, trapping and *in situ* observation of LC droplets in a single experiment has not been reported before.

**Fig. 1:**
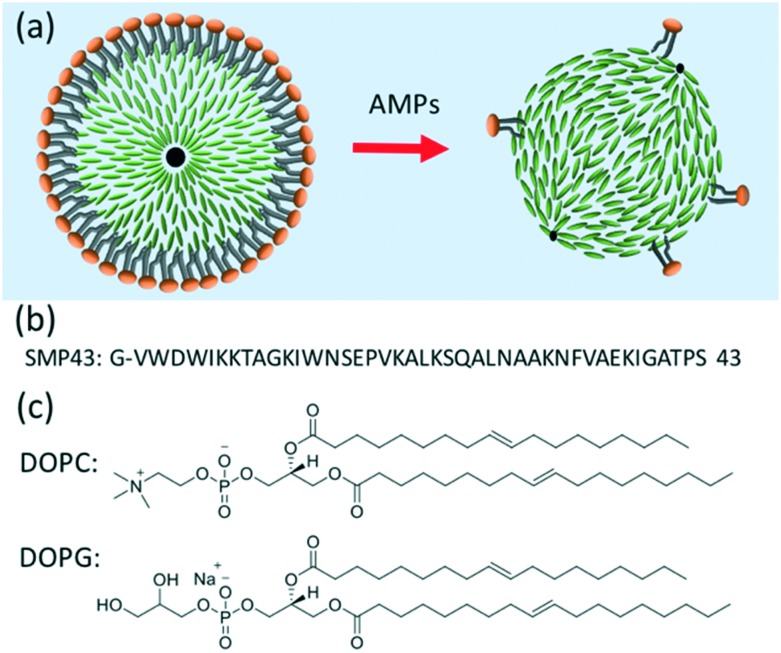
(a) Schematic diagram showing the bio-sensing mechanism. The lipid coating on the liquid crystal droplet initially induces a radial director geometry. Exposure to the AMP removes the lipid coating, inducing a planar surface alignment and bipolar droplet geometry. LC molecules: green rods. (b) Amino acid sequence of Smp43. (c) Molecular structure of 1,2-dio-leoyl-*sn*-glycero-3-phosphocholine (DOPC) and 1,2-dioleoyl-*sn*-glycero-3-phospho-rac-(1-glycerol) sodium salt (DOPG) lipids used for the monolayer lipid coating on the LC droplet.

Here, we are using Smp43 as a model peptide to develop LC based biosensors for the detection of AMPs utilizing a lipid coated liquid crystal droplet and the mechanism shown schematically in Fig. 1. Monodisperse lipid-coated liquid crystal droplets were produced using a microfluidic approach and confined in a trap structure that allowed gradients of AMPs to be flowed across the LC droplets in a controlled way. Smp43 (ref. 33) was used at different (micromolar) concentrations and the switching of LC droplets from radial to bipolar configurations was demonstrated by *in situ* polarized optical microscopy. Our results show the potential of lipid-coated droplets for AMP detection, which will be a useful tool in antibiotic drug discovery screening programs.

## 2 Experimental details

### 2.1 Materials

The nematic liquid crystal mixture E7 was purchased from Synthon Chemicals GmbH & Co. KG, Germany. Smp43 (MW = 4654.4, purity = 97.7%) was synthesized using solid-phase chemistry and was purchased from Think Peptides, Oxford, UK. 1,2-Dio-leoyl-*sn*-glycero-3-phosphocholine (DOPC), 1,2-dioleoyl-*sn*-glycero-3-phospho-rac-(1-glycerol) sodium salt (DOPG), and HEPES were purchased from Sigma-Aldrich. The molecular structure is shown in Fig. 1(c). The premium glass microscope slides were from Fisher Scientific (Pittsburgh, PA). Sylgard 184 silicone elastomer was purchase from Farnell, UK. All aqueous solutions were prepared with deionized water, using a Milli-Q water purification system (Millipore, Bedford, MA).

### 2.2 Preparation of lipid liposomes

The liposome solution was made by hydration and tip-sonication of dried lipid mixture (DOPC&DOPG 1 : 1 with 0.1 mol% Texas Red-DHPE) in 10 mM HEPES buffer (pH 7.5), as described previously.^34^ The Texas Red-DHPE was included to allow fluorescence imaging of the lipid-coated droplets, providing confirmation of the presence or removal of the lipid. 15% volume of glycerol was added to the liposome solution to increase the viscosity, optimizing the flow properties for microfluidic liquid crystal droplet production.

### 2.3 Microfluidic device fabrication

The PDMS microfluidic device fabrication follows the protocol reported previously.^32^ In brief, a silicon master with a SU8 pattern was fabricated using a MW2 laser direct writing system. The inverse PDMS copy of the silicon master, with punched holes for the inlets and the outlet, was plasma cleaned (100 W, O_2_ pressure 0.5 mbar, 1 min, Zepto Plasma Unit, Diener Electronic, Germany) and bonded to a cleaned glass plate. The resulting device was baked at 75 °C for 30 min and the channels were coated with a thin layer of PVA to make them hydrophilic.^32^

### 2.4 Lipid-coated liquid crystal droplet production

**Fig. 2:**
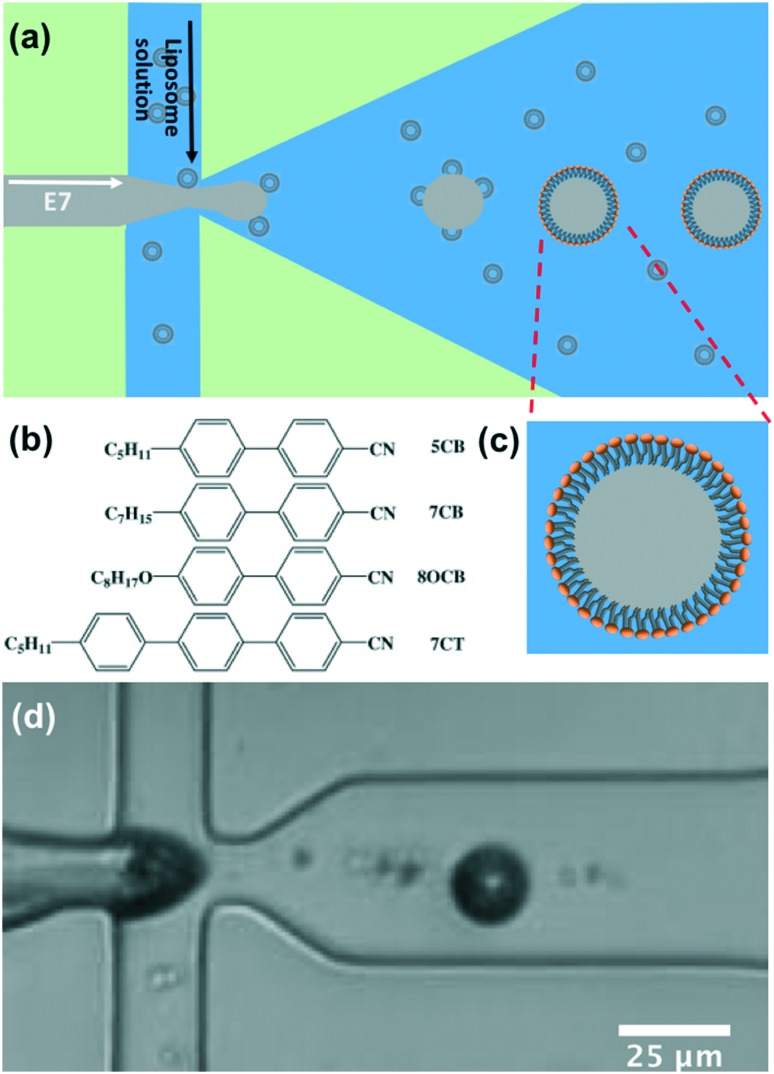
(a) Schematic of the microfluidic device used to form lipid-coated E7 droplets. (b) Molecular structure of the compositions in the mixture of E7. The weight percentages are 51%, 25%, 16%, and 8%, respectively. (c) Schematic diagram of the lipid coated LC droplet. (d) Image of the droplet formation in the actual microfluidic device, taken using a high-speed camera (videos can be seen at ESI‡ Movie S1). The liquid crystal (E7) is injected from the middle channel and the liposome solution is injected from two side channels.

Monodisperse lipid-coated droplets (diameter = 17 μm) were produced using a flow focus droplet microfluidic device.^35^ A schematic diagram of the droplet formation process in the device is shown in Fig. 2(a). The droplet formation device had two inlets. One inlet fed the two outer side channels, with buffer solution containing lipid in the form of small unilamellar liposomes. The middle inlet was used for the feeding of liquid crystal (E7). The E7 and liposome solutions were pumped into the device through the two inlets using two PHD ULTRA advanced syringe pumps (Harvard Apparatus, USA). The flow rate used for LC droplet generation was 0.075 μL min^–1^ for E7 and 10 μL min^–1^ for buffer with liposomes. E7 is a commercially available nematic LC material. It is a mixture of four different liquid crystal compounds, Fig. 2(b). E7 has been previously used in the thin film form for the study of the interaction between cationic antimicrobial peptides and lipid membranes.^7^ Compared to 5CB, which is frequently used in biosensor applications, E7 has a wider nematic range.^36^

The liquid crystal material (E7) entered the nozzle and was pinched-off by the buffer solution surrounding it due to the shear force.^35^ Lipid vesicles in solution adsorbed and ruptured at the hydrophobic LC/water interface to form a monolayer on the surface, Fig. 2(a) and (c). A movie taken by a high-speed camera (Photron SA5) recording the liquid crystal droplet formation process at the frame rate of 100k is attached as ESI‡ Movie S1. Fig. 2(d) is a frame from the movie showing the formation of the droplets.

### 2.5 Fluorescence microscopy

The fluorescence signal from the fluorophores included in the lipid layers was observed using an epifluorescence microscope (Nikon Instruments Europe B. V., Kingston, UK) equipped with a Texas Red filter block. Fluorescence images were captured using an Andor Zyla sCMOS camera, Orca-ER (Oxford Instruments plc, UK).

### 2.6 Polarized microscope observation

The liquid crystal droplets were suspended in buffer and held between two microscope cover slips or sealed in the microfluidic traps for polarized microscope observation. A Leica DM 2700P, Leica Microsystems Ltd polarized microscope equipped with a pair of linear polarizers and a Nikon camera (model D3000) allowed high quality images of the droplets to be recorded at magnifications of 100× to 500×.

### 2.7 On-chip Smp43 detection using a concentration gradient trap structure

**Fig. 3:**
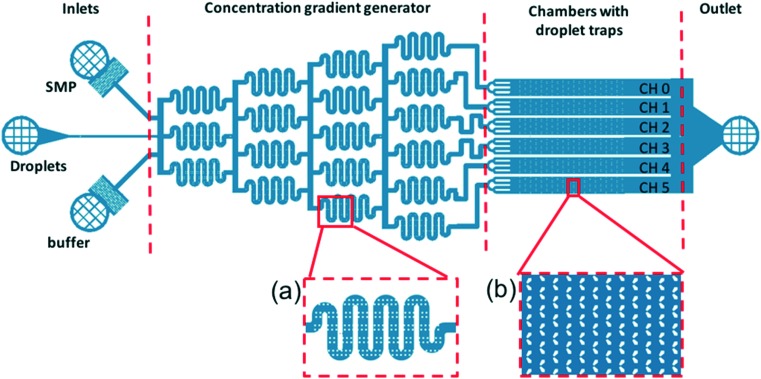
Trap structure with a concentration gradient generator used to evaluate the Smp43 detection by the lipid-coated LC droplets. The device has four main parts: inlets, concentration gradient generator, chamber with droplet traps, and outlet. (a) Expanded view of the serpentine channel and pillars in the channel to promote mixing; (b) expanded view of the trap region indicating how the droplets are trapped in the swallowtail features.

The detection of Smp43 using trapped lipid-coated E7 droplets required a specially designed trap structure that allowed droplets to be exposed to a known concentration gradient of the AMP, Fig. 3.

This structure is composed of four main parts. The inlet part has three inlets in parallel for the feeding of AMP (top inlet), LC droplets (middle inlet) and buffer (bottom inlet), respectively.

**Fig. 4:**
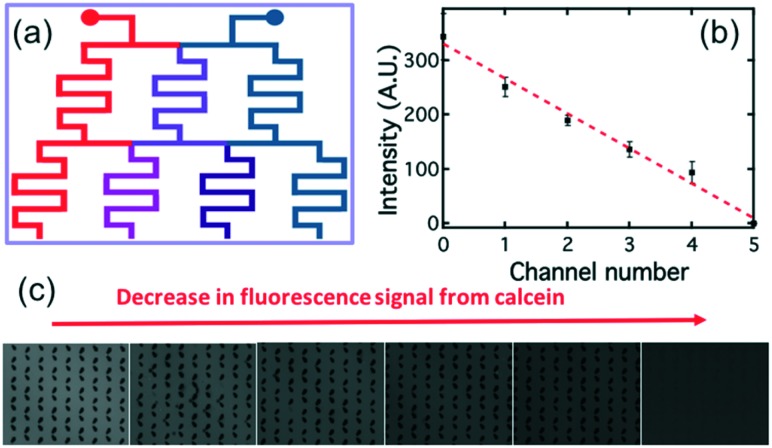
(a) Schematic showing the principle of the “tree shape network” design that generates a linear concentration gradient. (b) Measurements of the concentration gradient of calcein formed in the six trap chambers, at a flow rate of 0.2 μL min^–1^, measured by determining the fluorescence intensity of the calcein. The behavior can be described by the linear fit shown in red. (c) Fluorescence images taken for different trap chambers.

The “concentration gradient generator” part of the system operated using the classical “tree shape network” design, as shown in Fig. 3.^37^Fig. 4(a) gives a schematic diagram indicating the principle of such a design. For each stage, streams are split before flowing into the serpentine-shaped branch channels of next stage. All neighboring branch streams join together and give a new concentration after mixing in the channel, while the two outermost channels keep a constant concentration, the same as that of the two inlets. In the experiment, the two inlets are supplied with buffer solution and AMP; these materials then pass through the generator at suitable flow rates (ranging from 0.2 μL min^–1^ to 1 μL min^–1^) becoming thoroughly mixed in the serpentine structures. To test the linearity of the concentration gradient across the six trapping chambers, the fluorescent material calcein was passed into one inlet with buffer into the other. The fluorescence images of the trap chambers (as labeled in Fig. 3) are displayed in Fig. 4(b), when a flow rate of 0.2 μL min^–1^ was simultaneously applied to each of the inlets. Fig. 4(c) shows the linear concentration gradient of calcein was generated in these chambers. Linear concentration gradients could be obtained in the trap chambers for flow rates in the range from 0.2 to 1 μl min^–1^ (Fig. S3‡).

The trap regions consisted of six chambers with “swallowtail”-shaped features to trap droplets positioned in arrays offset by a half period. The region where the two ‘wings’ meet had a gap of ∼8 μm, designed to trap the ∼17 μm droplets. The swallowtail-shaped hydrodynamic trap structure has not previously been reported in literature^38^ and there are several advantages to this design compared with conventional trap structures. Firstly, the two ‘wings’ are designed to reduce the resistance of the gap to the flow. Secondly, the design reduces the difficulties of making patterns with small features (<10 μm) in a relatively thick SU8 layer (25 μm). Thirdly, this trap structure can trap larger droplets if the flow is in the opposite direction. Therefore, it can work as a dual-mode trap structure.

For the Smp43/LC droplet experiments, a buffer solution (10 mM HEPES, pH 7.5) was initially flowed through the device at very high flow rates (400 μL min^–1^) to remove any air from the device. Then, the solution containing the lipid-coated droplets was fed through the droplet inlet at a flow rate of 2 μL min^–1^ through the droplet inlet, keeping the other two inlets blocked. The droplet traps in all of the chambers were filled with LC droplets in 10 minutes. The droplet inlet was then blocked, and the buffer solution and the Smp43 solution (at a concentration of 6 μM) were allowed to flow through the other two inlets simultaneously at a flow rate of 0.4 μL min^–1^. The careful design of the microfluidic structure ensured that different concentrations of Smp43 (6, 4.8, 3.6, 2.4, 1.2, and 0 μM, respectively) flowed through the different trap chambers.

The device was viewed using the polarizing microscope to observe any change in the director field of the lipid-coated droplets under continuous flow of the Smp43. Images were taken every 5 min until the experiment was complete.

## 3 Results and discussion

**Fig. 5:**
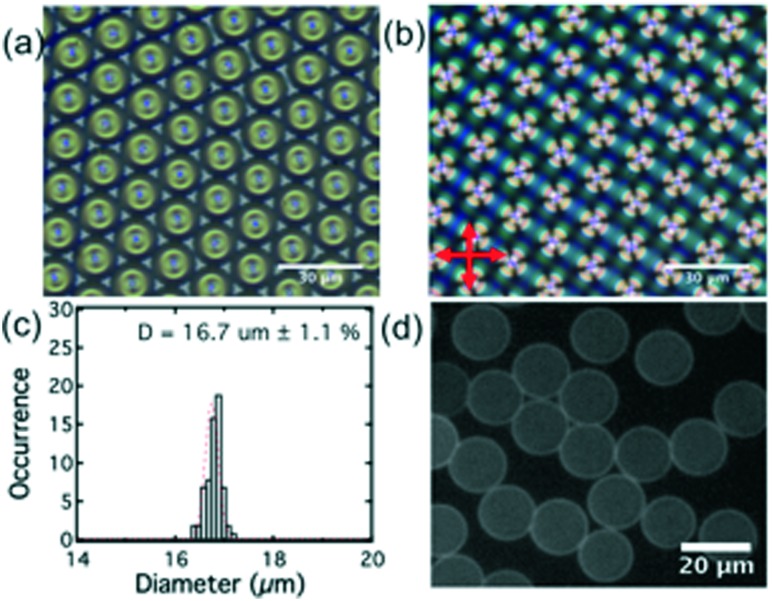
(a) Bright field image of E7 droplet arrays taken in the transmission mode; (b) image of droplet arrays taken in transmission mode polarizing microscopy (with crossed polarizers); (c) histogram of the size distribution of E7 droplets; (d) fluorescence image of the lipid-coated DOPC/DOPG (PC/PG 1 : 1) E7 droplets after excess liposome is washed out of the solution. The standard deviation (HWHP) for the fluorescence intensities of the droplets is 11.3%.

Lipid-coated LC droplets were produced using the microfluidic methods outlined above. The bright-field image of the droplets taken in the reflection mode, Fig. 5(a), shows that these droplets pack with almost perfect hexagonal symmetry because of their highly uniform size. The +1 defects are clearly visible at the droplet centers, representative of the radial configuration of the LC.

Observation of these droplets *via* polarizing microscopy (Fig. 5(b)) shows as a Maltese cross birefringence pattern, indicating that all of the droplets have a radial director configuration. This is expected due to the strong homeotropic anchoring induced by the lipids at the interface.^2^ These droplets were heated above the nematic/isotropic transition temperature and held there for several minutes and then cooled to produce a uniform radial alignment at room temperature. The as-produced lipid coated E7 droplets (without thermal treatment) are not in a perfect radial configuration, as shown in the ESI‡ Fig. S1, a phenomenon attributed to shear alignment experienced by the droplets during their production.

The histogram of droplet size distribution, Fig. 5(c), confirms excellent monodispersity. The peak was fitted with a Gaussian function, with a half-width of half peak (HWHP) of only ∼0.2 μm (∼1.1% of the droplet diameter). This compares favorably with most reported LC droplets generated by microfluidics.^39^ The average size of the droplets used in this study was 16.7 ± 0.2 μm.

The size of the LC droplets can be tuned by varying the relative flow rate of the buffer (*F*_W_) and the liquid crystal (*F*_LC_), as shown in the Fig. S4.‡ The diameter of the droplets decreased from ∼20.5 μm to ∼16.5 μm on increasing *F*_W_/*F*_LC_ from 4 to 1000, Fig. S4.‡ Outside this range, the formation of droplets became unstable due to the pressure balance between the buffer and LC channels.

During the formation of the E7 droplets, a monolayer of lipid was coated onto the surface, as shown in Fig. 2(a). This stabilized the droplets in solution by preventing their aggregation and coalescence. For all the LC droplets, the lipid monolayer was visible under fluorescence microscopy because of the inclusion of fluorescently labeled lipids (Texas Red-DHPE) in the lipid monolayer, Fig. 5(d). No defects could be observed in the lipid monolayer under ×400 magnification. At the lipid concentration of 1 mg ml^–1^ used in this study, the lipid monolayer will fully cover the surface of LC, confirmed through careful comparison of the fluorescence signal of lipid monolayers with that of a supported lipid bilayer reported previously.^20^ Similar to lipids in giant unilamellar vesicles (GUVs) and supported lipid bilayers, the lipids at the liquid crystal/aqueous interface are mobile. We have studied the mobility of lipids at the LC thin film/aqueous interface using the fluorescence recovery after photobleaching (FRAP) method. The diffusion coefficient of lipids was found to be 2.3 μm^2^ s^–1^, Fig. S2.‡ This value is slightly higher than that reported for a supported lipid bilayer (1.5 μm^2^ s^–1^),^34^ but is comparable to the previously reported value for lipids at the surface of LC thin films.^20^ The mobility of the lipids in the monolayer at LC/aqueous interface is a good indication that they are in a state associated with normal biological function. Thus, they are an appropriate receptor for the detection of AMPs.

The lipid mix used in this study contains 50% negatively charged DOPG lipid (a good bio-mimic for bacterial membranes), which further increases the stability of the E7 droplets due to the electrostatic repulsive force between the droplets. Experiments determined that the droplets did not show noticeable change in size or number per unit volume when kept for more than a week.

For the *in situ* observation of the radial to bipolar switching of droplets on exposure to Smp43, we deployed our novel trap structure with its concentration gradient generator on a single chip, as shown in Fig. 3. The thermally treated droplets, initially 100% in the radial state, were used for all the experiments in this study.

**Fig. 6:**
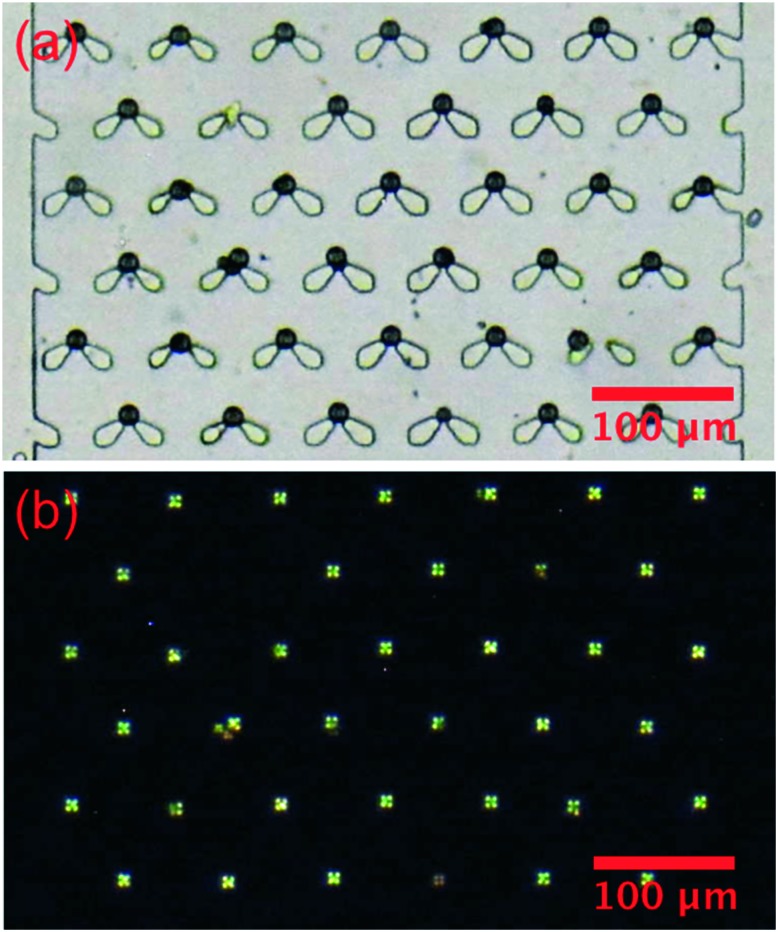
Images of the PC/PG coated E7 droplets trapped in the swallowtail shaped trap structures in one of the chambers. (a) Bright field image; (b) image taken using polarized microscopy in transmission.

The LC droplets were passed through the specially-designed trap structure and held at the narrow head region of the trap features, Fig. 6. The trapping was extremely efficient, reaching nearly 100% in 10 min at a flow rate of 2 μl min^–1^. Fig. 6(a) is a bright field image of the trapped droplets, showing both the droplets and traps. Fig. 6(b) is an image of the same sample taken under transmission mode polarized microscopy. The cross patterns indicative of a radial droplet structure are clearly visible. Of course, the non-birefringent traps are not visible under this mode.

After the LC droplets were trapped in the device, the droplet inlet was blocked and the buffer inlet and Smp43 inlet were connected, feeding the device with buffer and Smp43 at a flow rate of 0.4 μl min^–1^. The concentration of SMP43 was 6 μM at the Smp43 inlet. After the concentration gradient generator, the concentration of Smp43 in the different trap chambers was 6, 4.8, 3.6, 2.4, 1.2, and 0 μM (from top to bottom in Fig. 3).

**Fig. 7:**
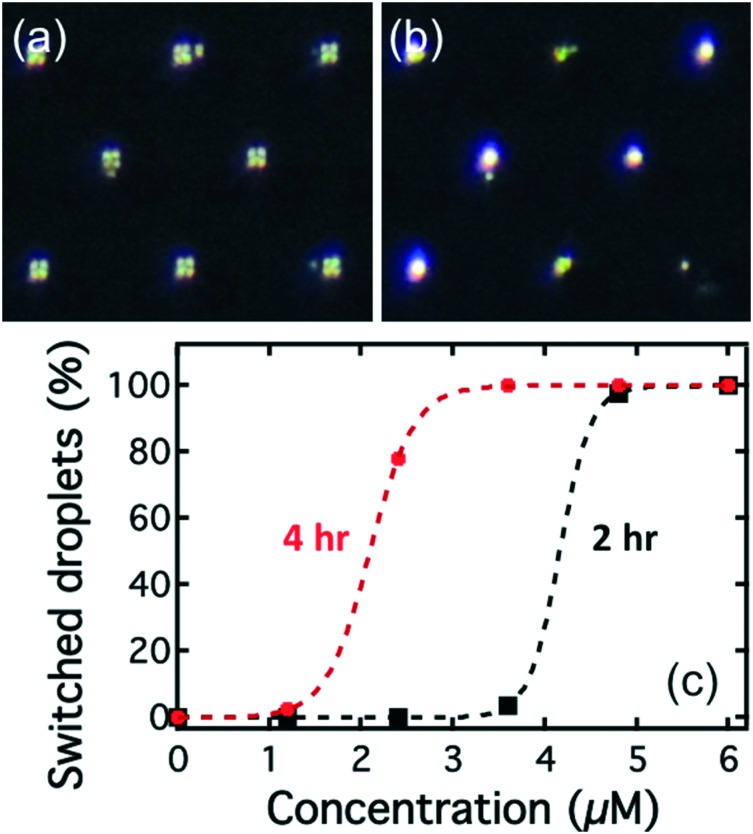
(a) and (b) are typical images of arrays of droplets in traps viewed under polarizing microscopy before and after Smp43 (4.8 μM) treatment, respectively. The images clearly show the switching from radial to bipolar states ((a) and (b) respectively). (c) Percentage of switched LC droplets, after two hours (black squares) and four hours (red dots) as a function of Smp43 treatment at different concentrations. The dashed lines are fits to these two sets of data using Sigmoid function. In this case, the concentrations that show full switching are 4.8 and 3.6 μM for the two-hour and four-hour treatment, respectively.

Initially, all the droplets had the Maltese cross birefringence pattern indicative of a radial structure, Fig. 7(a). As the droplets are exposed to the Smp43, they begin to change appearance with the crossed patterns first transforming to an irregular texture before exhibiting a birefringence pattern characteristic of a bipolar structure, Fig. 7(b). A video recording of this process can be found in ESI‡ Movie S2.

The percentages of the droplets that had switched director orientation at two hours in the different chambers are shown as the black squares in Fig. 7(c). After 2 hours, almost all of the droplets rinsed with Smp43 at concentrations of 4.8 and 6 μM had switched from the radial to the bipolar state (98% and 100% respectively), Fig. 7(c). However, only 4% of droplets exposed to the 3.6 μM Smp43 had switched and for lower concentrations of Smp43, no switching was detected. The data suggested that the detection limit for Smp43 by the lipid-coated E7 droplets was 4.8 μM at two hours. After four hours of exposure (red circles in Fig. 7(c)), more droplets in the chambers with Smp43 concentration of 2.4 and 3.6 μM had switched to the bipolar configuration. The detection limit at four hours was 3.6 μM.

**Fig. 8:**
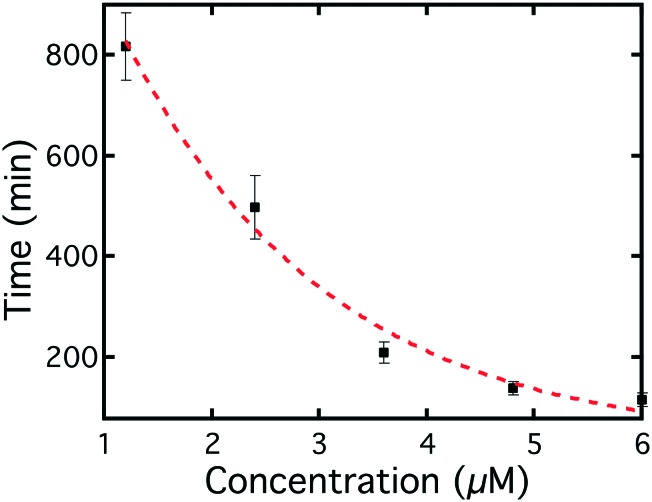
Average switching time of the droplets as a function of Smp43 concentration. The switching time showed a monotonic decrease with increasing Smp43 concentration. The images show clear radial and bipolar textures before and after exposure to the Smp43.

The average switching time for different Smp43 concentrations is shown in Fig. 8, as can be seen, the higher the concentration, the shorter the switching time. This is as expected since the higher the concentration, the faster the Smp43 can remove the lipids from the surfaces of the LC droplets. There was a distribution of switching times of the droplets in the same chamber, which was fitted to a Gaussian distribution; the peak position was chosen as the average switching time of droplets and the half width of the half peak as the error bar. The distribution was concentration dependent, with lower concentrations producing wider distributions of switching time, as indicated by the error bars in Fig. 8.

The interaction of Smp43 with supported lipid bilayers has previously been studied utilizing fast scan AFM and QCMD by our group.^15^ These results showed that Smp43 exhibits preferential membrane disruptive activity against bacterial-membrane mimics (PC/PG) compared to mammalian-membrane mimics (PC/PE).^15^

Direct confirmation of the removal of the lipid coating on the LC droplet surface by Smp43 treatment, was obtained *via* fluorescence microscopy. Fig. S5‡ shows the fluorescence intensity after ten hours' exposure to Smp43. As might be expected, the fluorescence signal was far weaker for the droplets in the chambers with a higher concentration of Smp43. Indeed, an 80% drop in the fluorescence signal for droplets treated with 6 μM Smp43 was observed, compared to the droplets treated in buffer solution without Smp43.

The switching process of the droplet director was more complicated than initially expected, as the droplets were observed to switch even in pure buffer solution after ∼8 hours rinsing, at the flow rate of 0.4 μl min^–1^. Indeed, when rinsed with higher flow rates in pure buffer solution the droplets switch faster suggesting that the shear force from the flow will also contribute to or trigger the switching from the radial to the bipolar state. This is understandable, as shear forces are well known to influence LC molecules at the surfaces.^40^ As there are two effects that can contribute to the droplet switching, one from Smp43 and one from the flow, care was taken to deconvolute the phenomena. The effect of the flow of the buffer is determined using the data obtained for the droplets in the chamber with no Smp43. The times shown in Fig. 8 are those corresponding to the switching time caused by Smp43 alone. Detailed analysis of how the phenomena are separated can be found in Fig. S6.‡ Interestingly, the droplet switching can be modeled theoretically, using a pseudo first-order process for the effect of Smp43 and a different first-order process for the effect of the buffer. This approach is as discussed in ESI‡ Model S1. The model suggests a linear dependence between the Smp43 concentration and the inverse of switching time, confirmed by the experimental results as shown in Fig. S7.‡

## 4 Conclusions

In this study, monodisperse DOPC/DOPG (1 : 1) lipid coated E7 droplets of the diameter of ∼17 μm have been produced using a microfluidic device in the flow-focus regime. These droplets showed a radial configuration once any initial effects due to shear alignment effects had been thermally annealed out. The radial droplet director pattern is readily observed as a characteristic Maltese cross using a transmission polarizing microscopy. The droplets have been used as reporter for the detection of Smp43 – an atypical α-helical model AMP.

A novel trap structure was designed to allow the study of the effect of Smp43 concentration as a function of time in a single experiment. The droplets were trapped in the separate chambers, while the gradient generator provided a linear concentration gradient of Smp43 across the different trap chambers. Under conditions of continuous rinsing with buffer and Smp43, the LC droplet director geometry was switched from radial to bipolar. The detection limit of Smp43 is at the μM level which is within the limit of detection for AMPS with Smp43 exhibiting antimicrobial properties between 0.9 and 28 μM.^33^ AMPs are currently being designed in their own right for the selective targeting of cancer cells as well as bacterial cells and our platform, with different lipid coatings could thus find application as a rapid ‘non-animal’ based screening platform to evaluate the efficacy of these new AMPs. Further, the novel trap structure will be useful for the study of bulk or interfacial reactions/interactions for all kinds of droplets *in situ*.

For real-world applications of biosensors, sensitivity, response time and selectivity are important factors to optimize. Our approach offers the opportunity to optimize all three of these important parameters. We have demonstrated the switching with a detection limit was at μM level, which is comparable to the study of LC thin film sensor for the detection of PGLa – an AMP from frog skin.^7^ The switching time demonstrated for our system, about 2 hours at 4.8 μM, is currently a bit slower than the desired operational time for a biosensor (a few tens of minutes).^3^,^41^,^42^ Further, the lipid coated LC biosensor has the potential to detect a variety of membrane interacting biomolecules in solution, such as bacteria toxins, AMPs, neurodegenerative proteins, cell signaling peptides or synthetic polymers with selectivity increased by modulating phospholipid composition.

## Conflicts of interest

There are no conflicts to declare.

## Supplementary Material

Supplementary movie

Supplementary movie

Supplementary information
