# Supplementary material for: Lipid coated liquid crystal droplets for the on-chip detection of antimicrobial peptides
Source: Lab Chip. 2019 Feb 20;19(6):1082–9. doi: 10.1039/c8lc01291a (PMC6484679; doi:10.1039/c8lc01291a)
Supplement: Supplementary file 3 [file LC-019-C8LC01291A-s003.pdf]

Electronic Supplementary Material (ESI) for Lab on a Chip.
This journal is © The Royal Society of Chemistry 2019

Support information for

Lipid coated liquid crystal droplets for on-chip detection of antimicrobial peptides

Peng Bao,[a] Daniel A Paterson[a], Patrick Harrison[b], Keith Miller[c], Sally Peyman[a], J Cliff Jones[a], Jonathan Sandoe [d], Stephen D Evans [*a], Richard J Bushby [*a], Helen F Gleeson [*a]

Support information 1- Experimental details:

Support information 2- Support information figures:

Support information 3- Model S1:

Support information 4 - Video:

Support information 1- Experimental details:

1. Preparation of lipid liposomes

The liposome solution was made by hydration and tip-sonication of dried lipid mixture (DOPC&DOPG 1:1 with 0.1mol% Texas Red-DHPE) in 10mM HEPES buffer (pH7.5), as described previously [1]. The Texas Red-DHPE was included to allow fluorescence imaging of the lipid-coated droplets, providing confirmation of the presence or removal of the lipid. The lipid mixture was dissolved in chloroform in a glass vial and dried under nitrogen flow for 1h. Buffer solution was then added and the vial was vortex-mixed for 2 minutes to produce a suspension of the lipids. The suspension is tip-sonicated for 30 min at 4 °C until it is clear. The resulting liposomes have a size of ~ 25-30 nm [1]. The liposome solution was normally kept at 4 °C and used within one week. 15 % volume of glycerol was added to the liposome solution to increase the viscosity, optimizing the flow properties for microfluidic liquid crystal droplet production.

2. Microfluidic device fabrication

To make the polydimethylsiloxane (PDMS) devices, a silicon master with a SU8 pattern was first fabricated. The SU8 patterns with a thickness of 25 μm were formed on a three-inch silicon wafer using a MW2 laser direct writing system (Durham Magneto Optics Ltd, Durham, UK). The SU8 pattern was then hard-baked at 210°C for 15 minutes to improve the mechanical properties of the SU8 master.

To make an inverse PDMS copy of the SU8/Si master, the PDMS monomer was first fully mixed with the curing agent at a weight ratio of 10:1. The master was placed at the bottom of a petri dish and the PDMS monomer mixture poured onto it to yield a thickness of ~5 mm. The system was then placed under vacuum for 30 minutes to remove any air bubbles. Finally, to crosslink the PDMS, the petri dish was placed in an oven at 75°C for 1 hour.

The PMDS layer was temporarily transferred to a glass plate and holes at the inlets and outlets made using a biopsy punch. The PDMS device was plasma cleaned (100W, O_2 pressure 0.5mbar, 1min, Zepto Plasma Unit, Diener Electronic, Germany) and bonded to a second cleaned glass plate. Applying a gentle pressure and baking in an oven at 75 °C for 30min forms a PDMS microfluidic device that was ready for use.

3 Lipid-coated liquid crystal droplet production

Monodisperse lipid-coated droplets (diameter = 17 μm) were produced using a flow focus droplet microfluidic device [2]. A schematic diagram of the droplet formation process in the device is shown in Figure 2(a). The droplet formation device had two inlets. One inlet fed the two outer side channels, with buffer solution containing lipid in the form of small unilamellar liposomes. The middle inlet was used for the feeding of liquid crystal (E7). The E7 and liposome solutions were pumped into the device through the two inlets using two PHD ULTRA advanced syringe pumps (Harvard Apparatus, USA). The flow rate used for LC droplet generation was 0.075 μL/min for E7 and 10 μL/min for buffer with liposomes.

Support information 2- Support information figures:

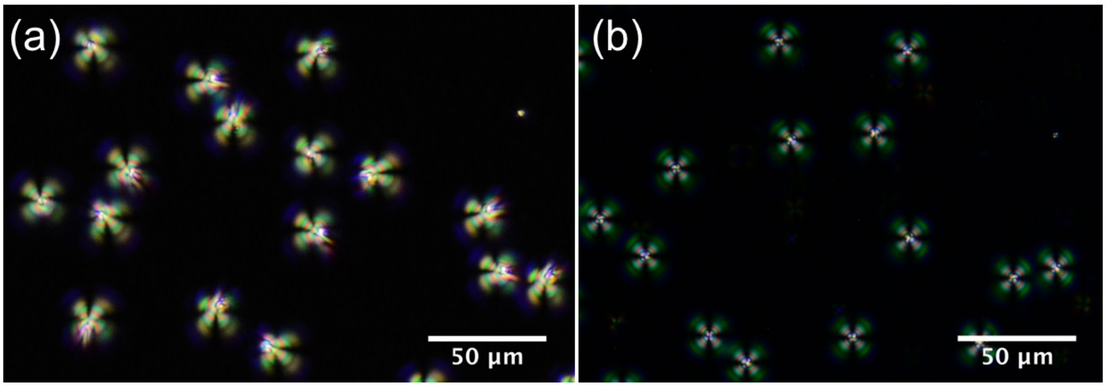

Figure S1. Images of (a) the as-produced PC/PG (1:1) coated E7 droplets and (b) the same droplets experience a thermal treatment at 75 °C for 20min, taken under crossed polarizer in transmission mode of polarized microscope.

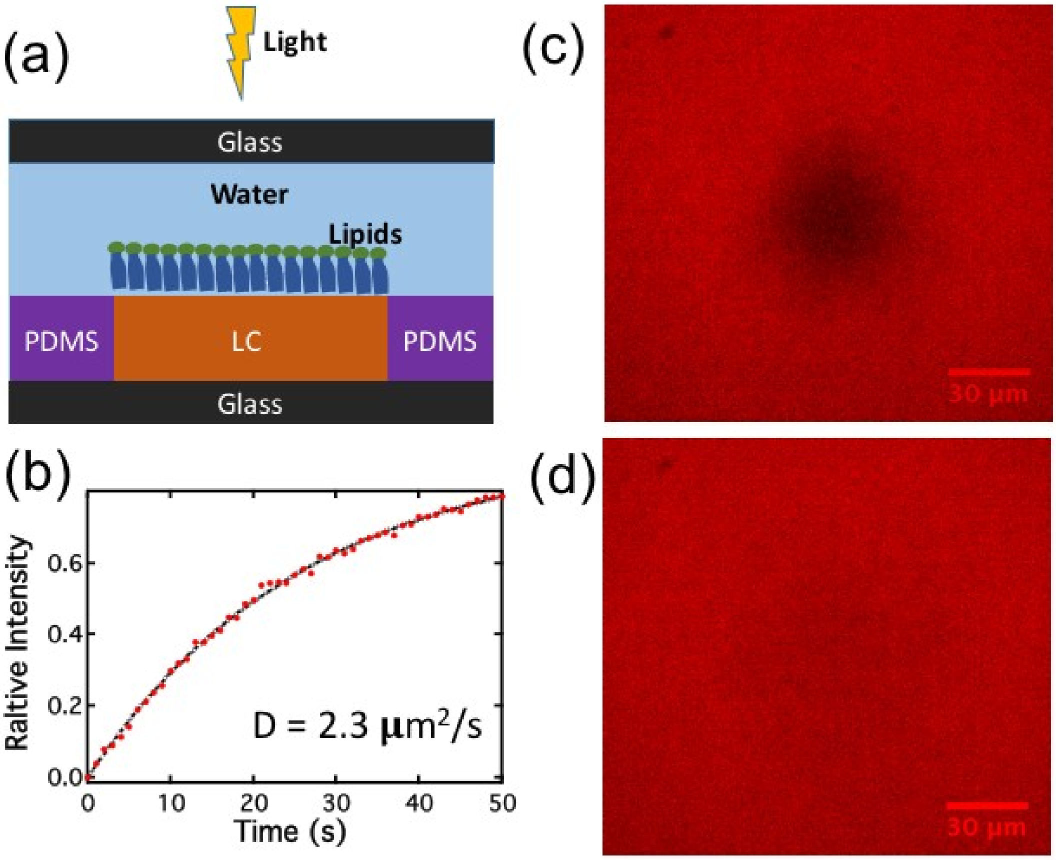

-

Figure S2. FRAP experiment for the lipid monolayer (DOPC/DOPG (1:1) with 0.1mol% Texas Red- DHPE) at E7 thin film/aqueous interface. (a) Schematics of the experimental setup. (b) Recovery curve of the fluorescence signal in the bleaching region. The diffusion coefficient is 2.3 µm^2/s. The fluorescence image of the lipid monolayer taken at (c) immediately after photobleaching; (d) 50 s after photobleaching.

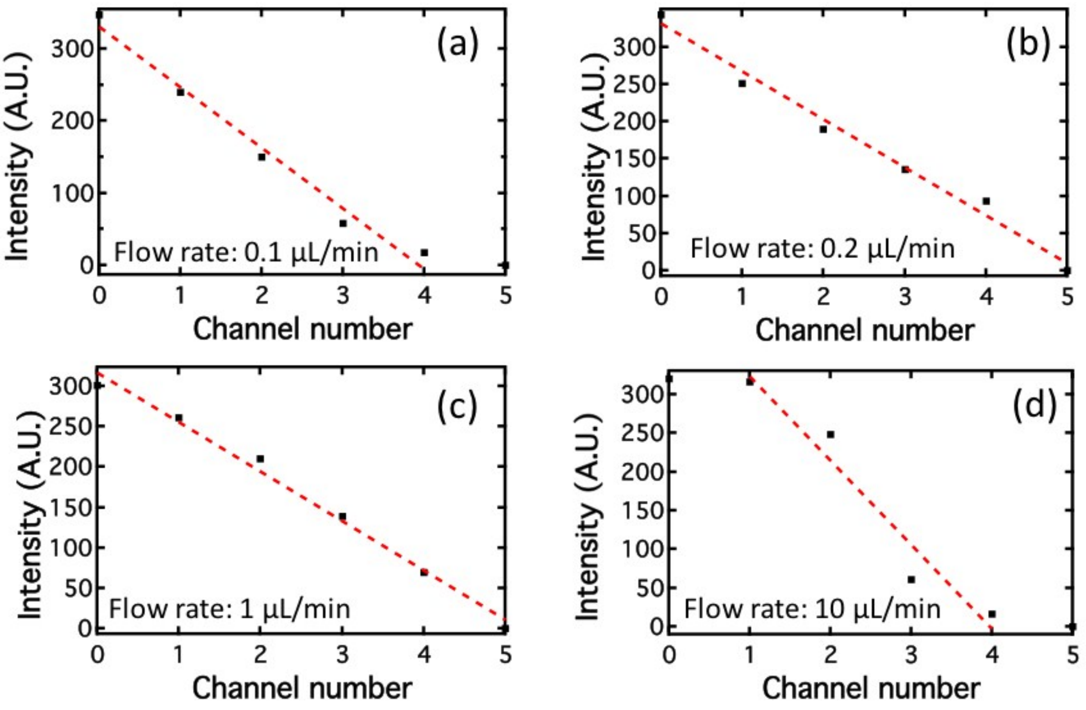

Figure S3. Measurements of the concentration gradient of calcein formed in the six trap chambers, at different flow rates of (a) 0.1 μL/min; (b) 0.2 μL/min; (c) 1 μL/min; (d) 10 μL/min, measured by determining the fluorescence intensity of the calcein. It can be seen that linear gradient can be formed in the flow rate range from 0.2 to 1 μL/min. We have used 0.4 μL/min for most of our experiments.

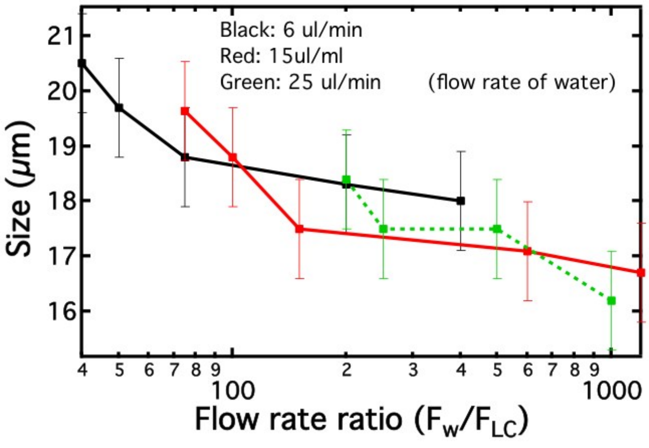

Figure S4. Size of the droplets produced as a function of flow rate ratio between buffer solution (F_W) and LC (F_{LC}). Three different flow rates of buffer solution have been used: 6, 15, 25 μL/min. The flow rate of LC varies under these conditions. The uncertainty in the droplet size shown in Figure S4 is bigger than that in Figure 3b because the size is determined from analysis of the high-speed camera images, which have a lower resolution than the still images used in Figure 3.

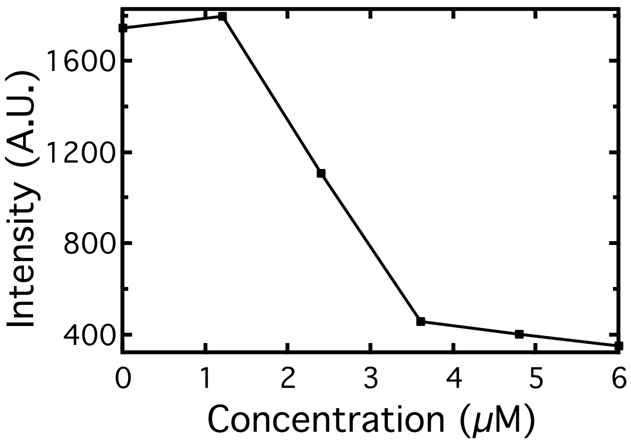

Figure S5. Fluorescence intensities for the droplets trapped in different chambers after ten hour's continuous exposure to SMP43 at the flow rate 0.4 µl/min at both the buffer and SMP43 inlets.

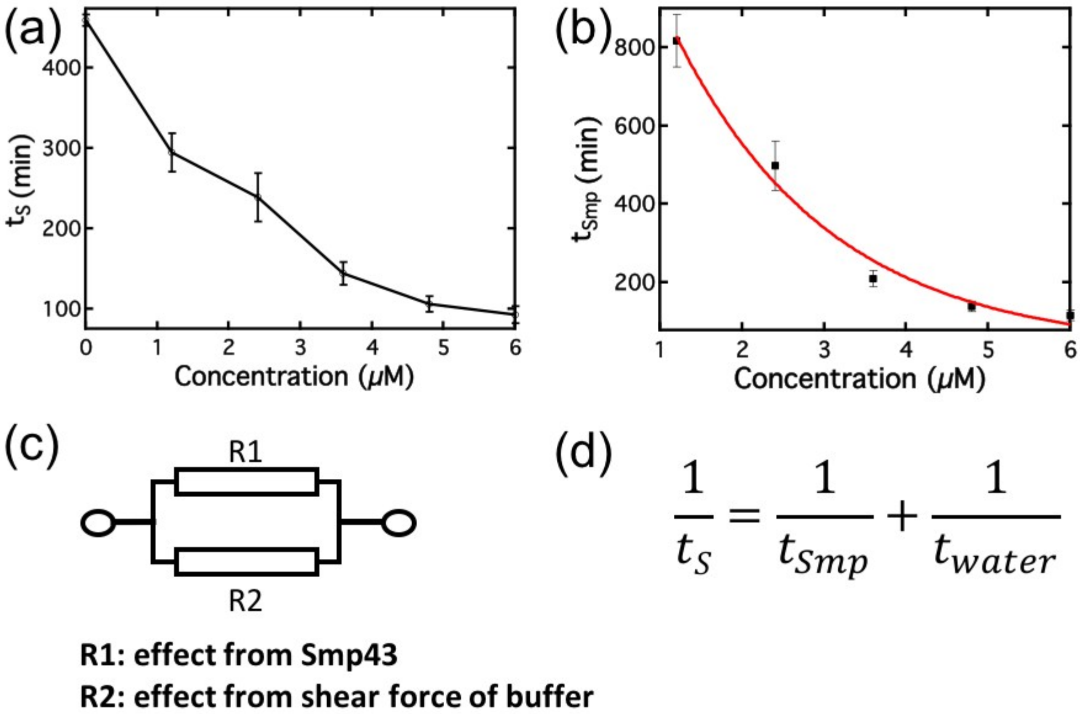

Figure S6. (a) Switching time (t_S) for different chambers with different SMP43 concentration; (b) Switching time of SMP43 (t_{Smp}), calculated from overall switching time (t_S) in (a) using the equation shown in (d); (c) Equivalent circuits for the combined switching process. The switching of droplets from radial to bipolar state is cause by two effects: disruption of lipids by Smp43 and the shear force from the buffer. These two processes are parallel process and therefore can be analogous to two resistors in parallel. (d) Equation used for the calculation of the switching time of Smp43, as shown in (b).

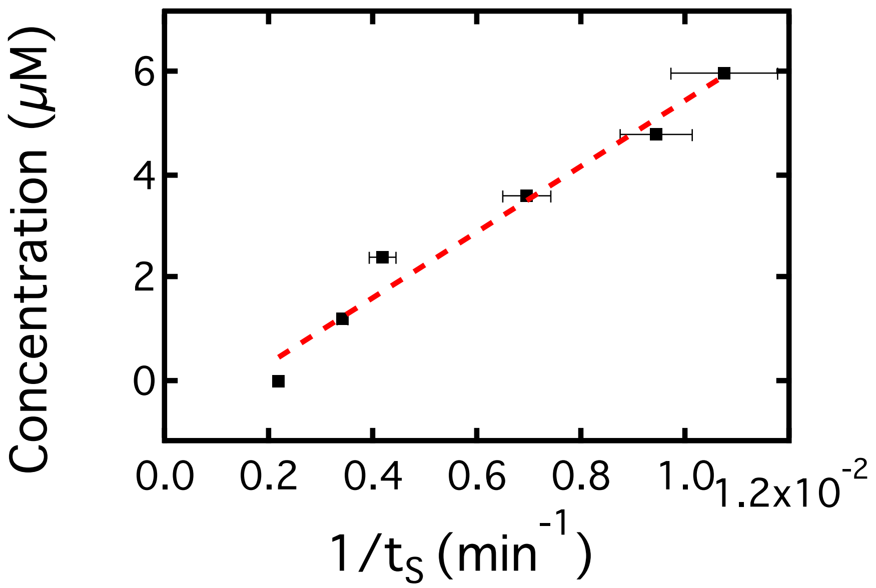

Figure S7. Dependence of concentrations of SMP43 as a function reverse of the switching times, which could be fitted to a line according the Model S1 shown below.

Support information 3- Model S1:

Model S1: The removal of lipids from the surface of the liquid crystal droplets.

The simplest model is to treat the removal/loss of the lipid from the surface as a combination of one process which is dependent on the concentration of venom [M] and one which is independent (by buffer).

Taking the first of these as second order (the simplest possibility)

$-d[L]/dt = k_1[M][L]$ where [L] = concentration of lipid

Or. If the concentration of venom is kept constant during the measurement then it is pseudo first order

$-d[L]/dt = k_{1M}[L]$ where $k_{1M} = k_1[M]$

If the other process is dependent only on [L] (is first order)

$-d[L]/dt = k_2[L]$

Overall

$-d[L]/dt = (k_{1M} + k_2)[L]$

Rearranging

$-d(\ln [L]) = \{k_{lm} + k_2\}\, dt$

Integrating and applying the boundery condition that at t = 0, $L = L_o$ (the initial lipid concentration on the surface) gives

$\ln\{[L]/[L_0]\} = -(k_{1M} + k_2)\, t$

If the director switches from radial to planar at a fixed value of $\ln([L]/[L_0]) = -C$ then the time $\{t_s\}$ at which this happens can be written:

$C = (k_{1M} + k_2)\, t_s$

Rearranging and substituting $k_{1M} = k_1[M]$ gives

$[M] = \{C/k_1\}\{1/t_s\} - \{k_2/k_1\}$

Hence a plot of [M] versus $1/t_s$ should give a straight line slope C/k_1 and intercept $-\{k_2/k_1\}$.

Support information 4 - Video:

Video S1. The formation of the PC/PG (1:1) coated E7 droplets using a microfluidic device, as mentioned in Figure2. Frame rate: 50k per second.

Video S2. The switch of the PC/PG (1:1) coated E7 droplets in the trap structure under the constant flow of SMP43. Images were taken every 5 min.

References:

1. P. Bao, M. R. Cheetham, J. S. Roth, A. C. Blakeston, R. J. Bushby and S. D. Evans, *Analytical Chemistry*, 2012, **84**, 10702-10707.
2. H. Suz-Kai, C. Cheng-Tso and L. Gwo-Bin, *Journal of Micromechanics and Microengineering*, 2006, **16**, 2403.
